# The Relationship Between Physical Activity and Problematic Internet Use in Turkish College Students: The Chain-Mediated Role of Self-Control and Distress

**DOI:** 10.1007/s11126-025-10133-x

**Published:** 2025-04-03

**Authors:** Fatih Gür, Ganime Can Gür

**Affiliations:** 1 Faculty of Sport Science, Department of Coaching Education, University of Pamukkale, Denizli, 20000 Türkiye; 2 Faculty of Health Science, Department of Psychiatric Nursing, University of Pamukkale, Denizli, 20000 Türkiye

**Keywords:** Chain mediation, College students, Distress, Internet addicton, Physical Activity, Self control

## Abstract

This study aims to investigate the impact of physical activity on problematic internet use and to reveal the mediating role of self-control and psychological distress in the path relationship between them, providing a theoretical foundation for reducing problematic internet use and promoting mental health among college students. This cross-sectional study was conducted with undergraduate students. Data were collected online using network sampling through the online survey platform Google Forms. Data were collected using the Demographic Data Form (DDF), Physical Activity Scale-2 (PAS-2), the Addiction Profile Index Internet Addiction Form (BAPINT), the Brief Self-Control Scale (BSCS), and the Psychological Distress Scale (K10-PDS). In order to evaluate the adequacy of the research model, path analysis, a component of structural equation modeling (SEM), was used using AMOS software. The structural model also exhibits a good fit, with x^2^ /df = 3.105, RMSEA = 0.081, GFI = 0.964, AGFI = 0.924, CFI = 0.948, TLI = 0.914, and IFI = 0.949. Physical activity could directly negatively predict the problematic internet use of college students. Self control and distress partially mediate the relationship between physical activity and problematic internet use, and the mediating pathways included “physical activiy-self control- problematic internet use”, “physical activity-distress- problematic internet use”, and “physical activity-self conrtol-distress- problematic internet use”, accounting for 12.6%, 33.7% and 4.7% of the total effect, respectively. The results of this study provide an important framework for understanding internet addiction in this population, supporting the I-PACE model.

## Introduction

With the rapid advancement of information technology over the last twenty years, internet access has grown even more common. The total number of internet users globally reached 5.35 billion in January 2024. This corresponds to about 66.2% of the world's population [1]. In particular, restrictions related to the COVID-19 pandemic and limited face-to-face contact between people have significantly affected people's ways of working and daily lives, shifting many activities from offline to online and rendering people inextricably linked to the internet [2–4]. Although it is known that internet use has important benefits in many areas such as education, communication, entertainment and access to information, excessive and uncontrolled use of the internet can result in major issues like problematic internet use (PIU) [5].

PIU is a condition characterized by uncontrollable and excessive internet use that occurs as a result of an individual's inadequate capacity to regulate internet use, leading to psychological, social, academic and occupational problems and negatively affecting daily life functionality [6, 7]. Although PIU has not yet been accepted as a diagnosis, the Diagnostic and Statistical Manual of Mental Disorders-5 (DSM-V) [22 and The International Classification of Diseases-11 (ICD −11) [8] have added new diagnostic criteria for Internet gaming disorder, a subtype of Internet addiction. This concept includes addiction-like symptoms, negative emotional states and lack of cognitive control. Numerous empirical researches have shown that PIU is requently associated with a range of psychopathologic and behavioral problems, such as anxiety, insomnia, paranoia, depression, alcoholism, poor social skills, strained interpersonal relationships, and even an increased suicide risk [9–11]. Furthermore, these adverse conditions have been reported to contribute to a decline in the regularity of physical exercise among university students [12]. In addition, university students are more prone to PIU behaviors due to factors such as good social adjustment, sufficient time, easy access to the internet and lack of self-control [13], [14, 15]. Therefore, it is crucial to recognize risk factors and the underlying causes in order to develop effective prevention and intervention programs for internet addiction.

Theoretical models of the underlying mechanisms in the development and maintenance of addictive behaviors are crucial for both research and clinical practice. The Interaction-Person-Affect-Cognition-Execution (I-PACE) model has recently emerged as an important theoretical model to explain internet addiction [16]. The model is made up of four components, referred to as P-A-C-E. Personality and psychopathological qualities are among the fundamental attributes of the individual that are referred to by the P component. Components A and C refer, respectively, to the individual's emotional and cognitive reactions to internal or external events. Lastly, executive functions like self-regulation and impulse control are referred to by the E component. Brand considers the formation and maintenance of internet addiction as a process in which individual characteristics affect executive functions through emotional and cognitive processes, with these interactions leading to internet addiction behaviors [17]. Consequently, within the framework of the I-PACE model, this study intends to examine the impact of physical activity, a personal resource, on PIU, as well as whether negative cognitive and emotional responses to environmental stressors (e.g., distress) and executive functions (e.g., self-control) play a mediating role in the relationship between personal resources and PIU.

### Relationship Between Physical Activity and PIU

Physical activity is a vital component of a healthy lifestyle and plays a crucial role in the prevention and management of behavioral addictions and mental illness [18]. Current research suggests that physical activity may alleviate internet addiction (IA) by boosting the activation of neuromolecular mechanisms such as brain-derived neurotrophic factor (BDNF), noradrenaline and 5-hydroxytryptamine [19], [20, 21]. These neurochemicals can enhance the structure and function of synapses between neurons and promote the development of new neurons. As a result, this condition may compensate for and repair the damage caused by IA to brain function [19, 22]. A large number of studies have revealed a significant negative relationship between physical activity and internet addiction [23], [20, 24], [25]. Students who regularly participate in physical activity, for instance, typically have better mental and physical health and are less likely to be addicted to the internet (Y. [26]). Additionally, it has been found that high levels of physical activity directly and negatively affect internet addiction symptoms in university students, as well as problematic situations in each dimension (compulsion, withdrawal symptoms, tolerance, interpersonal health issues, and time management) [27].

### The Mediating Role of Self-Control

Self-control is a component of inhibition control, which is one of the basic executive functions. The cognitive processes, known as executive functions, are what enable a person to control their actions in order to achive specific objectives. Self-control, to put it more simply, is the capacity of an individual to control their thoughts, feelings, and behaviors despite the urges and impulses they encounter [28]. The limited self-control theory proposes that people have a limited capacity for self-control, that this capacity consumes certain resources, and that a lack of self-control can lead to undesirable behaviors or harmful habits such as addiction [29]. A meta-analysis study has shown a negative connection between self-control and internet addiction [30]. According to the I-PACE theory, a reduction in individual executive control and inhibition control leads to a weakening in the pursuit of motivation and suppression of desires, resulting in addictive behaviors [16]. Decreased inhibition control, in addition to being a vulnerability factor for addictive behaviors, is a moderator of the relationship between emotional responses to specific triggering stimuli and the behavioral decisions made as a result of these responses [17]. Therefore, it can be said that lack of self-control is a risk factor for internet addiction.

Meta-analysis studies have shown that physical activity has a positive effect on the executive functions, which form the basis of self-control [31, 32]. Moreover, other research have demonstrated that physical activity interventions (e.g. aerobic training, walking) are positively related to improvements in brain regions connected with cognitive regulation [33], [34]. Therefore, physical activity can improve self-control and thus reduce PIU. Studies conducted in adolescents and university students have indicated that self-control partially mediates the association between physical activity and PIU [27], [25], [35, 36].

### *The Mediating Role of Psychological Distress*

Psychological distress is a state of emotional distress linked to anxiety, depression, stress and general mood disorders. According to the I-PACE theory, individuals experiencing psychological distress tend to use the internet to regulate and cope with negative emotions such as stress, anxiety, depression [17]. The instant rewards offered by the internet (e.g. social media likes, achievements in games) can help people temporarily avoid these distressing feelings. This, in turn, may lead to automatic activation and potentially addictive behaviors [17, 37]. A growing number of studies have shown a positive correlation between psychological distress and PIU [38, 39], [40].

The mechanisms underlying the beneficial effects of physical activity on mental health are multifaceted. Physical activity contributes to improved mood and enhanced stress coping abilities by promoting the release of endorphins, neurotransmitters, and other neurochemicals [41]. Additionally, physical activity supports neuroplasticity in the brain, improving cognitive function and emotional regulation [42]. Some studies have shown that negative emotions such as depression and anxiety are closely related to cognitive dysfunction [43], [44], and that physical activity can have positive effects on cognitive function [14, 15]. Physical activity can therefore increase mental capacity by supporting cognitive processes. This enhanced cognitive function allows a person to better manage negative thoughts and cope more effectively with negative emotions. This situation helps people to develop healthier coping strategies by reducing the risk of excessive and escapist use of the Internet. Indeed, evidence from studies suggests that psychological distress mediates the relationship between physical activity and internet addiction [40], [25].

### The Chain Mediating Roles of Self-Control and Psychological Distress

In summary, physical activity, self-control and distress are antecedent variables of PIU, and both self-control and psychological distress have indirect effects in the relationship between physical activity and PIU [27], [36, 40]. At the same time, previous studies have shown that lower self-control is associated with increased psychological distress, and that self-control is a predictor of distress [45], [46]. Self-control is a factor that protects mental health, and according to the ego depletion process model, self-control leads to changes in one's motivation and attention, resulting in the emergence of more positive emotions [47]. Research has shown that tendencies toward excessive and insufficient control are associated with higher levels of depression, while anxiety symptoms increase with insufficient control [48], [49, 50]. Therefore, self-control plays a positive role in reducing psychological distress. Previous studies and the theoretical models have confirmed that individuals with low self-control show less inhibition when performing inhibitory control tasks, leading to excessive consumption of psychological resources and increased psychological distress. As a result, it is stated that this situation will affect internet addiction behaviors [17], [51], [25].

Therefore, the physical activity’s role in reducing problematic internet use can be explained through multiple mechanisms. Firstly, it is known that physical activity helps individuals to better manage internet use by increasing self-regulation [40]. Secondly, regular exercise may prevent people from turning to internet use by reducing stress and anxiety [24], [25], [26]. Lastly, the social interaction promoting effect of physical activity may encourage individuals to participate more in offline activities and thus prevent them from becoming addicted to internet use [27].

### Present Study

Based on previous studies, this study aims to investigate the impact of physical activity on PIU and to reveal the mediating role of self-control and psychological distress in the path relationship between them, providing a theoretical foundation for reducing problematic internet use and promoting mental health among college students. Previous studies have primarily focused on the direct relationship between physical activity and various psychological outcomes; however, there is limited research that explicitly examines the link between physical activity and PIU. This study makes an important contribution to the literature by examining the impact of physical activity on PIU, as well as the mediating role of self-regulation and psychological distress within this relationship. In particular, by illuminating the dynamics of both self-control and psychological distress in the relationship with PIU, it offers an innovative perspective in this field. In the existing literature, comprehensive studies in which these variables are addressed together are limited. In this respect, the study both contributes to theoretical knowledge and offers practical implications for intervention programs. Therefore, the hypotheses of this study are as follows: H1) Physical activity could directly and negatively predict PIU. H2) Self-control mediates the relationship between physical activity and PIU. H3) Psychological distress mediates the relationship between physical activity and PIU. H4) Self-control and psychological distress play a chain mediating role between physical activity and PIU.

## Methods

### Design

This cross-sectional study aimed to investigate the chain-mediating effects of self control and psychological distress between physical activity and PIU.

### Participants

A convenience sampling method was used to release an electronic questionnaire to 324 college students in a university in western Turkey. The inclusion criteria for this study were as follows: 1) older than 18 years, 2) affiliation to the included institution as a student, 3) sufficient competence in understanding Turkish and expressing oneself, and 4) expressing willingness to participate in the research. Exclusion criteria for participants included 1) not meeting the inclusion criteria and 2) giving irregular responses to the questions (e.g. repeated selecting the same option). In this study, a total of 336 questionnaires were collected, and 12 invalid samples were eliminated through descriptive statistics. Finally, 324 valid questionnaires were obtained, with an effective rate of 96%. The average age of the participants was 21.26 ± 3.11 years. In terms of sex, 63 (19.4%) participants were men and 261 (80.6%) were women.

Before starting the study, the sample size was determined using a tool developed by [52]. First, the number of observed (8) and latent variables (1) were entered into the tool, the effect size was set to small (0.1), the desired probability was set to 0.05 and the statistical power level was set to 80%. It was determined that a minimum sample size of 87 would be required. Given that SEM typically requires a large sample size, the researchers aimed to collect as much data as possible. Therefore, 324 participants met the minimum sample size requirement.

### Data Collection

Data were collected online using network sampling through the online survey platform Google Forms (https://www.google.com/forms/about/). Questionnaires were prepared using this platform and an online link was created. The link to the online survey was sent to undergraduate students through social media platforms (WhatsApp, Facebook, and Instagram) and they were asked to share it in their circles. This study was conducted on the basis of voluntary participation among undergraduate students studying at a university. It took approximately 10 min to answer all questions.

The process for obtaining informed consent from participants was included in the questionnaire. When the participants accessed the online survey, the first page included information about the aims of the study, that the survey was anonymous, that the results obtained would only be used for scientific research, that it would not pose any risk to their daily lives, and that participation in the study was completely voluntary. After the participants read the document and confirmed that they agreed to participate in the study, they were able to continue with the survey questions. No financial incentives or rewards were offered to the participants. The data collection process was carried out through online questionnaires and the participants filled out the questionnaires through the link sent to them.

Data were collected using the Demographic Data Form (DDF), Physical Activity Scale-2 (PAS-2), the Addiction Profile Index Internet Addiction Form (BAPINT), the Brief Self-Control Scale (BSCS), and the Psychological Distress Scale (K10-PDS).

### Measures

#### Demographic Data Form (DDF)

This form was developed by the authors to collect information about the socio-demographic characteristics of the participants. The form includes questions about age and gender.

#### Physical Activity Scale-2 (PAS-2)

The FAS-2 developed by Pedersen et al. [53] was adapted into Turkish by Gür [54]. This scale is a self-report scale for measuring physical activity level and sedentary behavior in adults. The scale consists of 9 items. It determines sedentary behavior at work, during transportation, and in leisure time, as well as the level of physical activity at different intensities: mild, moderate and vigorous. To estimate an individual's daily and weekly physical activity levels, the metabolic equivalent (MET) of each item is calculated. The daily or weekly physical activity level for each item on the scale was calculated by multiplying the MET value by the time reported by users. MET values were determined as follows: sleep, 0.9 MET; watching television, reading, and listening to music, 1 MET; sedentary work, 1.5 METs; standing and walking, 2.0 METs; mild intensity leisure activities, 3.0 METs, active transportation, 4.0 METs; heavy work, 5.0 METs; moderate and high intensity leisure activities, 5.0 and 6.0 METs respectively. To calculate the daily physical activity level of the person, divide the totals for mild, moderate, and vigorous physical activity (items 5, 6, and 7) by 7, and add this figure to the sums of other items. If the total daily time is less than 24 h (1440 min), the unaccounted time is added to the light physical activity category. If a value above 24 h is calculated, the extra time is subtracted from the light physical activity category. In addition, the test–retest reliability coefficient of Gür's [54] scale version is 0.81.

#### Addiction Profile Index Internet Addiction Form (BAPINT)

The BAPINT was developed by Ögel and colleagues [55]. The scale is a 5-point Likert-type self-assessment scale consisting of 18 items, ranging from "never" (0) to "almost always" (4). Looking at the validity and reliability studies of the scale, the results of the exploratory factor analysis for validity show that the scale has a structure consisting of 3 sub-dimensions, explaining 57.03% of the total variance. In terms of criterion-related validity, the correlation coefficient between BAPINT and the internet addiction scale was found to be 0.81. The area under the ROC curve was found to be 0.97. At the cut-off point of 2 points for BAPINT, the sensitivity is 0.90 and the specificity is 0.90. The Cronbach's α coefficient was found to be 0.88, and the test–retest reliability coefficient was 0.85 [55]. The total Cronbach’s alpha for this scale in this study was 0.905. The CFA model demonstrated a satisfactory fit [normed chi square (x^2^/df) = 2.879, comparative fit index (CFI) = 0.912, goodness of fit index (GFI) = 0.900, incremental fit index (IFI) = 0.913, root meansquare error of approximation (RMSEA) = 0.076, standardized root mean square residual (SRMR) = 0.063]. In line with the recommendations of Schumacker and Lomax [56], this scale was found appropriate for the sample studied.

#### The Brief Self-Control Scale (BSCS)

Developed by Tangney et al. [57] and adapted into Turkish by [58], the BSCS consists of 13 items and two dimensions. Each item is scored between 1 (does not apply to me at all) and 5 (completely applies to me). A high score on this scale indicates a high level of self-control, while a low score indicates a low level of self-control. The exploratory factor analysis conducted to determine the factors of the BSCS revealed a two-factor structure explaining 41.65% of the total variance. The dimension measuring impulsive behaviors is called impulsivity and was found to explain 22.95% of the total variance. Items 1, 2, 7, and 8 of this scale are included in the self-discipline subdimension. The dimension measuring control behaviors is called self-discipline and was found to explain 18.70% of the total variance. Items 5, 9, 10, 12, and 13 of this scale are included in the impulsivity subdimension. The total Cronbach's α coefficients for the scale, as well as for the self-discipline and impulsivity subscales, were calculated as 0.83, 0.81, and 0.87, respectively [58]. The total Cronbach’s alpha for this scale in this study was 0.748. The CFA model demonstrated a satisfactory fit [normed chi square (x^2^/df) = 2.612, comparative fit index (CFI) = 0.915, goodness of fit index (GFI) = 0.955, incremental fit index (IFI) = 0.917, root meansquare error of approximation (RMSEA) = 0.071, standardized root mean square residual (SRMR) = 0.051]. In line with the recommendations of Schumacker and Lomax [56], this scale was found appropriate for the sample studied.

#### Psychological Distress Scale (K10-PDS)

Developed by [59] at Harvard Medical School with the support of the U.S. National Center for Health Statistics. The Turkish validity and reliability were conducted by Altun and colleagues [60]. The scale consists of 10 items. The response options are based on a five-point Likert scale, ranging from always (5), most of the time (4), some of the time (3), rarely (2), to never (1). The scale has no subdimensions or reverse-coded items. The minimum score is 10, and the maximum score is 50. Higher scores indicate more psychological distress. In the Turkish reliability study conducted by [60], the Cronbach's alpha coefficient of the PDS was found to be 0.95. The total Cronbach’s alpha for this scale in this study was 0.929. The CFA model demonstrated a satisfactory fit [normed chi square (x^2^/df) = 3.162, comparative fit index (CFI) = 0.968, goodness of fit index (GFI) = 0.936, incremental fit index (IFI) = 0.968, root meansquare error of approximation (RMSEA) = 0.082, standardized root mean square residual (SRMR) = 0.035]. In line with the recommendations of Schumacker and Lomax [56], this scale was found appropriate for the sample studied.

#### Data Analysis

The analyses of this study were conducted using IBM SPSS Version 23.0 and AMOS 24.0 software. First, to determine the suitability of the dataset for analyses and whether it meets the basic assumptions of mediation effects, missing data, outliers, normal distribution, and multicollinearity issues were examined using SPSS 23.0 [61]. Univariate outliers were identified using z-scores, while multivariate outliers were determined using Mahalanobis distance values. According to the relevant literature, data with z-scores between −3 and + 3 are considered to be normally distributed [62]. Therefore, 12 data points were excluded from the dataset, and analyses were continued with 324 participants. As part of the analysis of multivariate outliers, Mahalanobis distance was calculated, and no data points were excluded as outliers. In the analysis conducted to determine whether the scale scores were normally distributed, the skewness and kurtosis coefficients of the scores were examined. These values are expected to be between −1 and + 1 [63]. Skewness and kurtosis criteria were met by each scale used in this study. Multicollinearity was checked for tolerance and variation inflation factors. In the literature, it is stated that the tolerance value should not be less than 0.1 and VIF values should not be greater than 10 [64, 65]. The tolerance value calculated within the scope of the research was found to be 0.911 for each of the independent variables and 1.098 for the VIF value. According to these values, it is seen that there is no multicollinearity problem between the variables.

After determining the suitability of the dataset, frequency and percentage values for descriptive variables were analyzed using SPSS 23.0. Descriptive statistics (mean, standard deviation, minimum and maximum values, Cronbach's alpha coefficient) for the variables of physical activity, internet addiction level, self-control, and distress were calculated. Pearson Product-Moment Correlation Analysis was conducted to evaluate the relationships between the variables examined and to validate the assumptions for subsequent analyses. A correlation coefficient (r) of less than 0.30 indicates a weak relationship, between 0.30 and 0.50 indicates a moderate relationship, and greater than 0.50 indicates a strong relationship [66].

Then, a structural equation model (SEM) was created using AMOS 24.0 software and a chain mediation effect model was established to analyze the model fit. Maximum likelihood estimation was used to estimate model fit and evaluate the structural model [62]. Model fit was assessed using the χ2 (CMIN), normed χ2 (CMIN/df) ≤ 3, root mean square of approximation (RMSEA) ≤ 0.08, standard root mean residual (SRMR) ≤ 0.05, comparative fit index (CFI) ≥ 0.90, goodness of fit index (GFI) ≥ 90, Tucker–Lewis index (TLI) ≥ 0.90, incremental fit index (IFI) ≥ 90, and normed fit index (NFI) ≥ 90 [67].

Finally, model number 6 was chosen in the macro program process in SPSS, and the equation model was analyzed by mediating effect with physical activity as the independent variable, self-control and distress as the mediating variables, and PIU as the dependent variable. The chain mediation role of self-control and distress in the relationship between physical activity and PIU in college students was tested with Bootstrap method. The bootstrap sample size was set as 5000 and the default 95% confidence interval was used. Results are considered statistically significant if the confidence interval does not contain 0 [68]. To further increase the rigor of the study, the Harman single factor test was used to test the bias of the common method before data analysis [69].

#### Ethical Considerations

Ethical approval was obtained from the Ethics Committee of Pamukkale University Faculty of Medicine in accordance with the Declaration of Helsinki (ethical approval number: E- 60116787-020-548375). Before starting the study, the participants were informed about the purpose of the study with the text at the beginning of the online questionnaire and were asked to indicate their voluntary consent. Participants were reminded that they could withdraw from the study if they wished and were assured that their personal information would remain confidential.

## Results

### Common method Bias Analysis

The use of a survey in this study raises the possibility of common method bias. Therefore, Harman's single factor test was used to assess the presence of common method bias [69]. All dependent and independent variables used in the study were included in the factor analysis. The results showed that there were 9 factors with eigenvalues greater than 1 according to the exploratory factor analysis. The variance explained by the first factor was 26.38%, which is below the critical value of 40%. Consequently, it can be said that the data in this study are not affected by common method bias.

### Correlation AnalysisBbetween the Variables

Table 1 presents the descriptive statistics and correlation matrix of the variables. The Pearson correlation analysis showed that physical activity was significantly positively correlated with self-control (r = 0.137, p < 0.05), and negatively correlated with distress (r = −0.138, p < 0.05), PIU (r = −0.190, p < 0.05), addiction symptoms (r = −0.193, p < 0.05), the impact of internet use on life (r = −0.130, p < 0.05), cravings for internet use (r = −0.166, p < 0.05), and motivation to reduce internet use (r = −0.107, p < 0.05). The self-control was significantly negatively associated with distress (r = −0.144, p < 0.01), PIU (r = −0.267, p < 0.01), frequency of internet use (r = −0.182, p < 0.01), addiction symptoms (r = −0.291, p < 0.01), impact of internet use on life (r = −0.217, p < 0.01), cravings for internet use (r = −0.177, p < 0.01), and motivation to reduce internet use (r = −0.136, p < 0.05). The distress was significantly positively associated with PIU (r = 0.572, p < 0.01), frequency of internet use (r = 0.300, p < 0.01), addiction symptoms (r = 0.488, p < 0.01), impact of internet use on life (r = 0.507, p < 0.01), cravings for internet use (r = 0.408, p < 0.01), and motivation to reduce internet use (r = 0.407, p < 0.01). The PIU was significantly positively associated with frequency of internet use (r = 0.578, p < 0.01), addiction symptoms (r = 0.846, p < 0.01), impact of internet use on life (r = 0.759, p < 0.01), cravings for internet use (r = 0.792, p < 0.01), and motivation to reduce internet use (r = 0.686, p < 0.01). The frequency of internet use was significantly positively associated with addiction symptoms (r = 0.461, p < 0.01), impact of internet use on life (r = 0.220, p < 0.01), cravings for internet use (r = 0.306, p < 0.01), and motivation to reduce internet use (r = 0.164, p < 0.01). The addiction symptoms was significantly positively associated with the impact of internet use on life (r = 0.649, p < 0.01), cravings for internet use (r = 0.576, p < 0.01), and motivation to reduce internet use (r = 0.482, p < 0.01). The impact of internet use on life was significantly positively associated with cravings for internet use (r = 0.592, p < 0.01) and motivation to reduce internet use (r = 0.400, p < 0.01). The cravings for internet use was significantly positively with motivation to reduce internet use (r = 0.372, p < 0.01). The correlation between the main variables was at the significance level, which provided a good basis for the subsequent mediation effect test.

**Table 1:** Correlation analysis among major variables

Variable	1	2	3	4	5	6	7	8	9
1. Physical activity	1	0.137*	−0.138*	−0.190*	−0.102	−0.193*	−0.130*	−0.166*	−0.107*
2. Self-control		1	−0.144**	−0.267**	−0.182**	−0.291**	−0.217**	−0.177**	−0.136*
3. Distress			1	0.572**	0.300**	0.488**	0.507**	0.408**	0.407**
4. Problematic internet use				1	0.578**	0.846**	0.759**	0.792**	0.686**
5. Freguency					1	0.461**	0.220**	0.306**	0.164**
6. Symptoms						1	0.649**	0.576**	0.482**
7. Impact							1	0.592**	0.400**
8. Craving								1	0.372**
9. Motivation									1
M ± SD	2654.18 ± 941.42	24.45 ± 3.42	25.47 ± 8.40	1.92 ± 0.03	3.62 ± 0.87	11.50 ± 4.57	7.62 ± 5.28	2.63 ± 2.07	3.35 ± 2.06
Skewness	0.347	−0.881	0.410	0.252	−0.435	0.132	0.580	0.486	0.266
Kurtosis	1.025	2.838	−0.604	−0.319	0.760	−0.239	−0.005	−0.409	−0.612

^**^p < 0.01; *p < 0.05

### Testing for Mediation

**Fig. 1:**
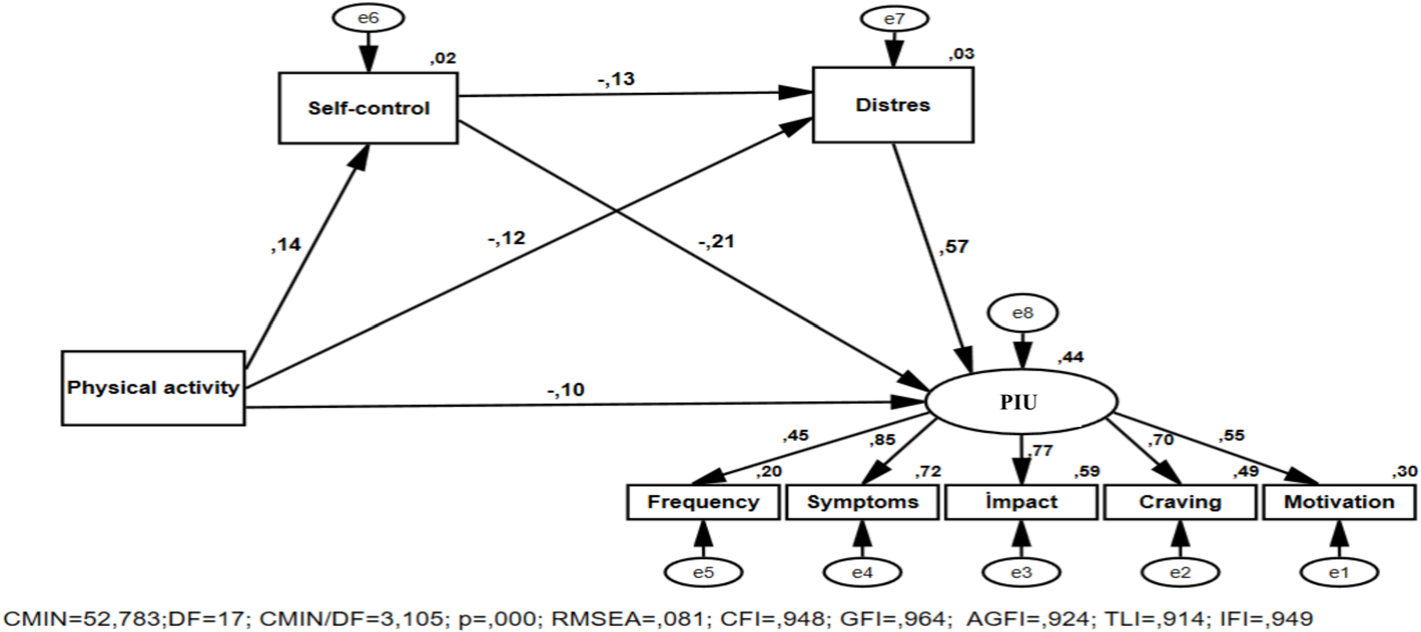
Research model and standardized factor loading values. RMSEA = root mean square error of approximation; CFI = comparative fit index; GFI = goodness of fit index; AGFI = adjusted goodness of fit ındex; TLI = Tucker-Lewis index; IFI = incremental fit index; PIU = Problematic internet use

The SEM was used to test the path relationship between variables (Fig. 1), and the fitting indexes of the model were as follows: × 2 /df = 3.105, RMSEA = 0.081, GFI = 0.964, AGFI = 0.924, CFI = 0.948, TLI = 0.914, IFI = 0.949, and, indicating that the model has a good fit and was suitable for mediating effect test. As shown in Tables 2, physical activity could directly and significantly negatively predict PIU (β1 = −0.209, SE = 0.000, p < 0.001). After adding the mediating variables of self-control and distress, the path coefficient used by physical activity on PIU decreased from β1 to β2, but the path coefficient still reached a significant level (β2 = −0.103, SE = 0.0006 p < 0.05). Physical activity could significantly positively predict self-control (β = 0.137, SE = 0.0002, p < 0.05), and self -control could significantly negatively predict PIU (β = −0.212, SE = 0.017, p < 0.001). Physical activity could significantly negatively predict distress (β = −0.120, SE = 0.0004, p < 0.05), and distress could significantly positively predict PIU (β = 0.568, SE = 0.009, p < 0.001). Self-control could significantly negatively predict distress (β = −0.126, SE = 0.135, p < 0.05).

**Table 2:** Standardized path coefficients between variables

Path			B	β	S.E	C.R	p	R^2^
Self-control	< –-	Physical activity	0.0004	0.137	0.0002	2.485	0.01	0.018
Distress	< –-	Self-control	−0.311	−0.126	0.135	−2.301	0.02	0.034
Distress	< –-	Physical activity	−0.001	−0.120	0.0004	−2.191	0.02	
Problematic internet use	< –-	Distress	0.076	0.568	0.009	8.104	< 0.001	0.436
Problematic internet use	< –-	Physical activity	−0.0001	−0.103	0.0006	−2.095	0.03	
Problematic internet use	< –-	Self-control	−0.070	−0.212	0.017	−4.067	< 0.001	
Frequency	< –-	Problematic internet use	0.343	0.445	0.052	6.585	< 0.001	0.198
Symptoms	< –-	Problematic internet use	3.433	0.851	0.347	9.869	< 0.001	0.724
Impact	< –-	Problematic internet use	3.575	0.767	0.378	9.453	< 0.001	0.589
Craving	< –-	Problematic internet use	1.276	0.699	0.142	8.986	< 0.001	0.489
Motivation	< –-	Problematic internet use	1.000	0.549				0.301

B: Unstandardized Coefficients; Β: Standardized Coefficients; SE: Standard Error; CR: Critical Ratio; R^**2**^: Squared Multiple Correlation

### Testing for Chain Mediation

The present findings also test that the chain mediating effect of self-control and distress in the relationship between physical activity and PIU (Table 3). The total mediation effect value of self-control and distress in the relationship between physical activity and PIU was −0.106 (accounting for the total effect ratio of 51.6%), and the Bootstrap 95% confidence interval did not include 0, indicating that the total mediation effect of physical activity and PIU was significant. The total mediating effect consists of three paths: (1) The indirect effect mediated by self-control was −0.029 (accounting for the total effect ratio of 12.6%), the Bootstrap 95% confidence interval did not include 0, indicating a significant mediation effect and supporting research hypothesis 2. (2) The indirect effect mediated by distress was −0.068 (accounting for the total effect ratio of 33.7%), the Bootstrap 95% confidence interval did not include 0, indicating a significant mediation effect and supporting research hypothesis 3. (3) The path with self-control and distress as chain mediators had an effect of −0.009 (accounting for the total effect ratio of 4.7%), and the Bootstrap 95% confidence interval did not include 0, indicating a significant chain mediation effect. This result confirms hypothesis 4. The total effect of physical activity on PIU was −0.209, with a direct effect of −0.103 (accounting for the total effect ratio of 48.4%) and was statistically significant. Hence, there is partial chain mediation of self-control and distress on the relationship between physical activity and PIU.

**Table 3:** Bootstrap analysis of the mediating effect test

Effect	Paths	Effect	BootSE	Boot95% CI		Effect size ratio
				Lower limit	Upper limit	
Direct effect	Physical activity → Problematic internet use	−0.103	0.000	−0.2033	−0.0069	48.4%
Indirect effect	Physical activity → Self-control → Problematic internet use	−0.029	0.011	−0.0486	−0.0056	12.6%
	Physical activity → Distress → Problematic internet use	−0.068	0.031	−0.1289	−0.0021	33.7%
	Physical activity → Self-control → Distress → Problematic internet use	−0.009	0.005	−0.0225	−0.0003	4.7%
Total indirect effect	Sum of all Indirect effects	−0.106	0.034	−0.1672	−0.0305	51.6%
Total effect	Total indirect effect + Direct effect	−0.209				100%

SE: Standard Error; Boot95% CI: 95% bias-corrected Confidence Interval

## Discussion

This study analyzed the effect of physical activity on PIU among university students and the chain mediating role of self-control and distress between this effect. To our knowledge, this is the first study to test the effect of physical activity on PIU through self-control and distress by applying the I-PACE model among Turkish college students. These results revealed that physical activity was negatively associated with the level of PIU among college students. Moreover, self-control and distress showed statistically significant mediating effects between physical activity and PIU. Overall, the results support the I-PACE model. According to this model, factors such as personal resources (e.g., physical activity), emotional and cognitive responses (e.g., distress), and executive functions (e.g., self-control) can create negative emotional responses depending on how the individual perceives the situation, which can lead to specific addictive tendencies. Accordingly, the results of this study will help to understand internet addiction more accurately and provide effective guidance for interventions for internet addiction among college students.

### The Direct Effect of Physical Exercise on PIU

Consistent with our hypothesis 1, the results showed that physical activity had a significant, direct negative predictive effect on PIU among college students, accounting for 48.4% of the total effect. This finding is in line with previous studies showing a negative relationship between physical activity and internet addiction [27, 70], [25], [26]. Additionally, in a meta-analysis study by Yan et al. [71] conducted with randomized controlled trials, exercise interventions were shown to be effective in reducing internet addiction levels and alleviating psychological symptoms among university students with internet addiction. We think that physical activity not only effectively improves individual health, but also its impact in reducing behavioral addictions is related to several factors.

First, according to the I-PACE model, physical activity, as a personal resource, can affect PIU through a combination of cognitive and emotional characteristics. Personal resources enhance an individual's coping mechanisms and allow them to cope stress more effectively. Therefore, physically active individuals may be less likely to engage in PIU because they feel less need to use the internet as an escape [17, 39]. This situation naturally reduces the amount of time students spend using the internet. Furthermore, physical activity positively affects the neurochemical balance in the brain by promoting the release of neurotransmitters such as endorphins, dopamine, and serotonin. These neurotransmitters play a critical role in regulating negative emotions such as stress, anxiety, and depression [19], [20]. There is considerable evidence in the literature that regular physical activity improves emotional regulation and enables individuals to cope better with negative emotional states [72], [25], [26]. These neurochemical changes associated with physical activity may mitigate or prevent the tendency to develop addictions during periods of stress or negative moods. It also has positive effects on cognitive functioning, as physical activity can improve abilities, attention, and decision-making [70, 73]. This cognitive and emotional development can reduce the individual's tendency to engage in PIU [16].

Furthermore, beyond these individual-level influences, the role of cultural and social factors in shaping the relationship between physical activity and PIU should also be considered [74–76]. In many cultural contexts, including Turkish university students, social expectations and norms regarding physical activity and internet use may play an important role in shaping behavior patterns. For example, societal attitudes towards sports participation, gender roles in physical activity participation, and the increasing digitalization of social interactions may influence the likelihood of students engaging in physical activity or excessive internet use [77], [23]; [78]. Research suggests that in cultures where physical activity is strongly encouraged and embedded in daily routines, individuals may be less prone to PIU due to the availability of alternative social and recreational activities [79]. [80]. These socio-cultural dynamics emphasize the importance of addressing cultural influences when designing interventions that promote physical activity as a strategy to reduce PIU.

Therefore, promoting healthy habits such as physical activity in the processes of preventing and managing PIU can help individuals improve both their mental and physical health, enabling them to manage internet use in a more balanced way.

### The Mediating Role of Self Control

Consistent with our hypothesis 2, our results showed that self-control played an indirect role as a mediating variable in the relationship between physical activity and PIU among college students, accounting for 12.6% of the total effect. This mediating role of self-control suggests that physical activity can reduce university students' PIU by improving self-control. According to the I-PACE model, executive control and inhibitory control skills are thought to be determinant in the development of addictive behaviors [17]. The findings obtained from the study show that physical activity significantly predicts self-control, with increased participation in physical activities increases individuals' self-control abilities. The finding that physical activity interventions improve executive functions and inhibitory control by activating the brain's prefrontal cortex supports the results of this study [31], [34]. Therefore, it is thought that physical activity, by enhancing the ability to control, can help individuals regulate their internet use and prevent the development of addictive behaviors. At the same time, the negative predictive relationship between self-control and PIU emphasizes the importance of self-control in reducing addictive behaviors, and this result coincides with the existing findings in the literature [27], [30], [25]. PIU behavior is a behavioral problem caused by individuals' lack of ability to control their internet use. Previous studies emphasize that individuals with higher levels of self-control are able to regulate their emotions and behaviors in a rational way and thus prevent the development of internet addiction symptoms. In addition, it is stated that this control capacity can be improved through physical activity [27], [81], [25].

These results confirmed previous studies that physical activity has a positive effect on self-control, but also suggested that physical activity can be used as a potential intervention strategy for the prevention or reduction of PIU. In this context, regular physical activity can be considered as an effective approach not only in terms of general health but also to improve individuals' ability to manage digital media use.

### The Mediating Role of Distress

Consistent with our Hypothesis 3, the results showed that distress played an indirect role as a mediating variable in the relationship between physical activity and internet addiction among college students, accounting for 33.7% of the total effect**.** This finding suggests that physical activity may reduce the risk of PIU among college students by reducing or preventing distress levels [40]. Additionally, it is observed that physical activity negatively affects distress, while distress positively predicts PIU. These findings provide an important framework for understanding the impact of psychological distress on internet addiction. Previous studies also suggest that distress plays a mediating role in the relationship between physical activity and internet addiction [40], [25], [49, 50].

The start of undergraduate education is a transitional period during which students are faced with the need to establish new interpersonal relationships, cope with academic pressures, resolve financial problems, deal with adaptation issues, cope with social pressures, face career uncertainty, and overcome the challenges of being away from home [82]. In this process, some individuals may experience negative emotions such as boredom, loneliness, anxiety, and depression. In this context, the internet can be used as a tool to cope with these challenges and may be preferred by students for social interaction, shopping, entertainment, guidance, and seeking advice [83]. However, uncontrolled and excessive use of the internet may increase the risk of PIU. Previous studies have shown that distress level is positively correlated with Internet addiction, and that individuals with high levels of distress tend to use the Internet as an escape [38, 39], [40]. The I-PACE model suggests that the impact of the biopsychological structure on the development of specific internet use disorders is explained through stress factors [16]. According to this model, individuals with high sensitivity to stress who use dysfunctional/impulsive coping strategies tend to respond with a desire to immediately change their mood when faced with stressful situations. This situation may increase the likelihood of the individual choosing a specific application or site, believing that the internet can reduce stress or having positive expectations related to the internet [17, 84].

In this context, physical activity, as a personal resource, has the potential to help individuals reduce stress levels and develop positive coping strategies. Studies have shown that regular physical activity can positively affect brain functions, increase the activation of the prefrontal cortex, and thus strengthen individuals' emotion regulation skills [42, 85]. These positive effects may reduce the risk of PIUs by reducing stress levels and replacing dysfunctional coping mechanisms with healthier strategies. The findings support this theoretical framework and highlight the mediating role of distress on PIU, emphasizing the potential of physical activity to reduce distress and lower the risk of PIU.

### The Chain Mediating Roles of Self-Control and Psychological Distress

Consistent with our Hypothesis 4, the findings showed that the chain mediation effect of self-control and distress in the relationship between physical activity and PIU was statistically significant. The total mediation effect accounted for 51.6% of the total effect, suggesting that physical activity may influence PIU through both self-control and distress. Additionally, the fact that both self-control and distress partially act as chain mediators, accounting for 4.7% of the total effect, suggests that physical activity affects self-control first, then distress levels, and ultimately reduces PIU. This situation is consistent with the existing literature that suggests psychological mechanisms such as self-control and distress play significant roles in behavioral addictions [51], [25].

One point to highlight in this study is the emphasis on the partial existence of chain mediation in the effect of physical activity on PIU. This suggests that the effect of physical activity on PIU cannot be explained entirely through psychological mechanisms (self-control and distress). Physical activity influences PIU both directly and indirectly through mechanisms such as self-control and distress. This indicates that while physical activity maintains its direct effect on PIU, mediating factors also play an important role. As a matter of fact, it is important to create intervention plans that not only promote physical activity but also include goals such as increasing self-control and reducing distress to prevent PIU. Apart from specific psychological mechanisms such as self-control or distress in the development and management of PIU, it is thought that different factors such as social support, academic stress, emotional intelligence may also affect PIU. It is suggested that future researchers conduct longitudinal studies to better understand the multiple interactions between physical activity and internet addiction, examine other potential mediating variables, conduct studies in different populations and contexts, and plan interventions targeting multiple mechanisms.

### Limitation and Future Direction

In this study, conducted using a cross-sectional research design and structural equation modeling, the relationship between physical activity and PIU has been examined. The study has some limitations. First, although this model reveals the path relationship between certain variables to a certain extent, it does not conclude a definite causal relationship. Therefore, future studies could focus on longitudinal follow-up or experimental intervention designs to establish the causal relationship. Second, the measures used in the study, which were based on the subjective experiences of the participants, have limitations. Future research could be designed to include objective data collection methods as well as subjective measures. Third, this study focused on young adults, which may limit the generalizability of the results. Therefore, it is recommended to conduct similar studies in populations with different age groups and demographic characteristics. This could allow to evaluate the effects of physical activity on internet addiction from a broader perspective. Fourth, socio-cultural factors affecting internet use and physical activity in the Turkish cultural context have not been addressed in detail. The influence of social norms, family expectations and traditional leisure time activities on university students' internet use and physical activity participation should be examined more comprehensively in future research. Finally, the majority of participants in this study were women (80.6%). For this reason, the findings of our study may not fully reveal the impact of gender differences and may limit the generalizability of the results in terms of gender. Future studies are recommended to create a more balanced sample to ensure gender equality.

## Conclusion

This study examined the impact of physical activity on PIU among university students and the mediating roles of self-control and distress in this relationship. The I-PACE model used to analyze these mediating factors provides an important framework for understanding internet addiction in this population. Our results revealed that physical activity was significantly and negatively associated with PIU, with both self-control and distress having statistically significant chain mediation effects. Thus, highlighting the potential for physical activity to also support students' internet use regulation skills. Likewise, the chain mediation effects of self-control and distress emphasize the importance of psychological mechanisms in behavioral addiction. The findings from this study provide evidence-based support for the inclusion of physical activity in intervention programs that increase self-control, reduce distress, and promote healthy lifestyle habits among college students to prevent and reduce PIU.

The findings of the study provide recommendations at both individual and institutional levels to reduce problematic internet use. At the individual level, educational programs can be organized to promote regular participation in physical activity through personalized exercise plans and training on digital tracking applications. At the institutional level, universities are encouraged to facilitate access to gyms, organize outdoor physical activities, and develop policies to regulate internet use. These interventions could not only reduce internet addiction but also enhance students' overall health and quality of life. In the future, applied studies examining the effectiveness of these approaches are recommended.

## Data Availability

The datasets generated during and/or analysed during the current study are available from the corresponding author on reasonable request.
